# Biochemical and haematological changes in HIV subjects receiving winniecure antiretroviral drug in Nigeria

**DOI:** 10.1186/1423-0127-20-73

**Published:** 2013-10-07

**Authors:** Bartholomew Okecuhukwu Ibeh, Olushola D Omodamiro, Urenna Ibeh, Josiah Bitrus Habu

**Affiliations:** 1 Department of Medical Biotechnology, National Biotechnology Development Agency, Abuja, Nigeria; 2 Department of Biochemistry, College of Natural and Applied Sciences, Michael Okpara University of Agriculture, Umudike, Abia State, Nigeria; 3 HIV/AIDS Unit, Emerging Health and Environment Initiative, Abuja, Nigeria; 4 Bioresource Development center Odi, National Biotechnology Development Agency, Abuja, Nigeria

**Keywords:** Haematological, HIV, Winniecure, ART, Biochemical, Nigeria

## Abstract

**Background:**

Hematological and biochemical abnormalities are among the most common clinicopathological manifestations of HIV patients on ART. Consequently, the development and assessment of indigenous antiretroviral drugs with minimal abnormalities becomes a necessity. The objective of this investigation was to assess potential haematological and biochemical abnormalities that may be associated with the administration of Winniecure ART in HIV patients undergoing treatment in Nigeria. Fifty (50) confirmed HIV positive ART naïve patients aged 36 ± 10 were observed for haematological and biochemical responses for 12 weeks. Haematological responses were assessed thrice at 6 weeks interval using coulter Ac-T differential analyser and biochemical indicators (bilirubin, creatine, urea, amylase, ALT, ALP, AST, albumin) assayed spectrophotometrically.

**Results:**

The biochemical parameters ALP (P < 0.05), ALT (P < 0.0001), AST (P < 0.001) and amylase (P < 0.05) slightly increased at the 12^th^ week, no significant change was observed in plasma creatinine and urea concentrations while albumin levels decreased non-significantly (P > 0.002). Haematological results showed consistent reduction of ESR, eosinophil, absolute and differential lymphocytes, granulocytes and total WBC in the test subjects throughout the assessment period. Conversely, haemoglobin, platelet and PCV increased significantly (P < 0.05). At the 12^th^ week thrombocytopenia (10.30%) and anaemia (76%) were reduced to 2% and 31% respectively while neutropenia (4.2 to 8%), leucopenia (26.8 to 30%) and lymphopenia (1 to 10%) increased. No cases of neutrophilia, lymphocytosis, eosinophilia and leukocytosis was observed.

**Conclusion:**

The drug has a reduced haematological abnormalities and normal kidney function was unaffected though there were signs of possible abnormal levels of hepatic enzymes beyond 12 weeks of treatment.

## Background

The human immunodeficiency virus (HIV) is one of the most important emerging infections of this century. It is probably one of the diseases with multiple impacts on persons, families, communities and the entire society. HIV is threatening especially in sub-Saharan African countries. In Nigeria for instance the prevalence rose from 3.8% in 1993 to 5.8% in 2001 [1]. Since the discovery and isolation of HIV in 1983, there has been significant progress in understanding the viral pathogenic proteins that cause the acquired immunodeficiency syndrome (AIDS) [2]. The subsequent development of HIV detection and quantification techniques is now used extensively both in research and routine clinical screening [3-6]. These techniques have provided a more complex understanding of the epigenetics, epidemiology and clinical features of the infection [7,8].

It is observed that the emergence of drug development programmes by government agencies, research establishments and pharmaceutical industries has led to many potent anti-HIV drugs called antiretrovirals [9]. Presently, a combination of these drugs known as highly active antiretroviral therapy (HAART) is recommended as a standard medication for the management of HIV infection. The advent of HAART, a cocktail of nucleoside and non-nucleoside analogues with potential to inhibit HIV reverse transcriptases and proteases [10] has lead to a reduced progression of the HIV infection to AIDS [11]. This however, results in improved quality of life of people living with HIV/AIDS [12].

It is undoubtedly observed that the HIV drugs are without some adverse medical effects with implicit safety limitations. Certain documented side effects of these drugs include diarrhea, nausea, abnormal distribution of body fats, anemia, neutropenia and cytopenia [13]. The need to consider the safety of any novel antiretroviral drug is of great importance and in line with centre for disease control and prevention (CDC) standpoint which advocated for evaluation of ART safety before being considered for administration. The present work therefore evaluates independently, possible hematological and biochemical abnormalities that maybe associated with Winniecure antiretroviral drug.

It is highly documented that the hematological consequences of HIV infection is dominated by peripheral blood cytopenia. This has become more common with the advent of antiretroviral therapy and related treatments for HIV-associated infections and malignancies [14,15]. Generally, in patients undergoing ART, anaemia may occur in approximately 60-70%, neutropenia in 50% and thrombocytopenia in 40% [16]. It is common that the incidence and severity of low cell count increases with advancing HIV disease. An exception to this is thrombocytopenia which maybe an early manifestation of HIV infection occurring in 3 to 12% of asymptomatic patients [17,18]. In addition myelo suppressive therapies and marno-infiltrating opportunistic diseases may further contribute to the development of cytopenia similar to the anaemia of HIV infection. An interesting clinical significance observed in Zidovudine (ZDV) and some drugs used for the treatment of opportunistic diseases is that they may produce therapy with HIV-induced neutropenia (a haematological abnormality) [19].

In view of the various questions raised by end users of antiretroviral therapy such as the safety and efficacy of the drug, a careful study of some possible abnormalities that may be associated with Winniecure an antiretroviral drug used in Nigeria was investigated.

## Methods

### Study population and ethics

Fifty confirmed HIV positive patients who were previously ART naive volunteered to take Winniecure antiretroviral therapy at a prescribed dose of 5 ml (1 tea spoonful) containing 250 mg Winniecure extract taken 3 times daily for 5 days and then 2 times daily for the period of this study (12 weeks). The study also had 100 HIV negative ART naive subjects attending clinic for the first time and this served as the study reference subjects or control group. All consenting subjects were recruited consecutively for the work. Blood samples were taken in accordance with the Nigerian National Ethics and Operational Guidelines for Research on Human subjects (NNEOGRHS). Part of the blood was collected into EDTA container to give blood-anti-coagulant concentration of 2 mg/ml of blood with sterile syringe (aseptic precautions were taken). The samples were collected at intervals of 6 weeks for the whole period of this study. A total of 150 subjects with an average age of 36 ± 10 for HIV^+^ ART subjects and 40 ± 52 for the control group were recruited for the study.

The inclusion criteria for enrolment of the study subjects were specified as follows; subjects must be either males or females aged 17 years and above, have confirmed laboratory evidence of HIV infection, accept to abide by the prescribed dosage of the drug, have history of no previous antiretroviral therapy (ART naive) and have CD4 cell counts between 50 and 350 cells/μl. Exclusion criteria include those with recent blood transfusion.

A structured questionnaire was filled by the subjects in order to obtain demographic data and clinical history and assistance was given on request. Prior to initiation of treatment, the HIV serostatuses of the patients were reconfirmed. Plasma samples from each of the patients were tested using double rapid tests kits (Cappillus and Unigold: Trinity BioTech, Ireland) and an ELISA method (Genie II HIV-1 & 2 kit). The tests used have different antigenic compositions.

### Haematological analysis

Parameters such as Hb, PVC, Total WBC, Differential WBC, platelet count and ESR were evaluated each time samples were collected from the subjects. Samples for ESR were analyzed using Westergren method [20]. Blood (2 ml) was diluted in 0.5 ml Trisodium Citrate solution using the Westergren pipette that was filled to a zero mark and mounted on a stand with the time adjusted to exactly 1 hour for the red cell to sediment. The column of the sedimented red cells was read at exactly 1 hour. Results were reported in mm/hour. Other haematological parameters; Hb, PCV, total WBC, differential WBC and platelet counts were analyzed with a haematology analyzer (Beckman Coulter AcT Diff. Hematology Analyzer). A suspension of blood cells was passed through a small orifice simultaneously with an electric current which introduces an impedance change in the orifice determined by the size of the cell. The system counts the individual cells and provides cell size distribution. The number of cells counted per sample is approximately 100 times greater than the usual microscope count which reduces the statistical error by a factor of approximately 10 times.

### Abnormalities

Certain abnormalities were determined in the subjects on antiretroviral treatment. Here anemia was defined as haemoglobin <13 g/dl (men) and <12 g/dl (women) while leucopenia as total WBC count less than 4000 cells/μl. Total platelet count <150 × 10^3^/μl was considered as thrombocytopenia. However, neutropenia was judged as absolute neutrophils/granulocytes count <1000 cells/μl and lymphocytopenia considered at absolute lymphocyte count of <800 cells/μl. Lymphocytosis, an increase in the number of lymphocytes was considered when the absolute lymphocyte count was greater than 4000 cells/μl. Neutrophilia indicates an elevated absolute granulocyte count above 7000 cells/μl while leukocytosis is defined as a total WBC more than two standard deviations above the mean, i.e. a value of greater than 11,000 cells/μl in adults. Also eosinophilia was evaluated at eosinophil count above 450 cells/μl. Absolute CD4 cell count determination was done by flowcytometric analysis.

### Biochemical analysis

Biochemical indicators were assessed at the first and last visits only. Commercially available kits were used to assay for the following serum enzymes: ALT, AST and amylase. Urine creatine (RANDOX) and urea (BioVision) were also determined using test kits. Bilirubin was measured directly by use of bilirubin oxidase [21] while albumin concentration was obtained by BCG (Quantichrom™ BCG Albumin Assay kit [DIAg-250] at 620 nm. This method is used daily in clinical laboratory measurement in Nigeria and the results are comparable to those of electrophoresis. Manufacturer’s procedure for each test was followed accordingly. Spectrophotometer was used to measure absorbance and results calculated correspondingly. Normal ranges were reported based on the results of the test kits used.

### Statistical analysis

All data analysis was done using SPSS version 17 statistical software package. Data was presented as Means ± Standard deviation (SD) and calculations done using the Student’s t-test and one-way ANOVA. Differences were considered to be of statistical significance at an error probability of less than 0.05 (P < 0.05).

## Results

### Study population

There was no significant difference between the ages of the HIV^+^ HAART group (36 ± 10) and that of the reference group (40 ± 52). A non-significantly higher (P > 0.05; 52%) number of HIV^+^ females and a significant increase (P < 0.05; 66%) in married couples amongst the HIV^+^ ART group were observed. However, 88% of the HIV^+^ ART subjects had CD4^+^ cells within the range of 200-350 cells/μl while 12% were between 50-200 cells/μl (Table 1).

**Table 1:** The baseline characteristics of the study population indicating CD4 count grouping, ART duration, marital status, age and gender

**Characteristics**	**ART**	**Reference/ control**	**P-value**
	**(n = 50)**	**(n = 100)**	
**Age (years; mean ± SD)**	36 ± 10^a^	40 ± 52^a^	0.084
**Gender (Female/Male; n{%})**	26{52}^a^/24{48}^a^	49{49}/51{51}	0.092
**Marital status (Married/Single; n{%})**	33{66}^a^/17{33}^b^	48{49}/52{52}	0.039
**Length of HAART (Weeks)**	9	NA	
**CD4 count**			
**Subjects; n (%)**			
1.50-200 cells/μl,	6, (12)	1100 ± 82 cells/μl	
11.200-350 cells/μl	44, (88)		

Comparison of data was done at P < 0.05, data on gender, marital status and CD4 cell count (measured as cells/ μl) was presented as absolute numbers (n) and percentages (%) while age was shown as mean **±** standard deviation in years. NA indicates none applicable. Data with different superscript indicates statistical significance.

### Haematological analysis

Table 2 summarizes the mean ± SD of some haematological parameters of HIV^+^ ART patients on Winniecure therapy before treatment (1^st^ assessment) and subsequent appointments at 6 weeks interval otherwise referred to as visits in this study.

**Table 2:** **Haematological parameters of HIV patients on Winniecure ART from the 1**^**st **^**assessment visit (before treatment) to the last visit ( 12**^**th **^**week)**

**Parameters**	**1**^**st **^**visit**	**2**^**nd **^**visit**	**3**^**rd **^**visit**	**Reference value**
ESR (mm/1 hr)	41.46 ± 31.01^a^	40.33 ± 35.36^a^	32.12 ± 15.49^b^	19.6 ± 40
Total WBC (×10^3^ cells/μL )	6.23 ± 1.96^a^	5.70 ± 0.06 ^b^	5.65 ± 0.73^b^	8.40 ± 1.5
Absolute lymphocytes (×10^3^ cells/μL )	3.04 ± 1.44^a^	2.48 ± 0.68^b^	2.30 ± 0.51^b^	3.88 ± 2.5
Absolute granulocytes (×10^3^ cells/μL )	3.03 ± 1.46^a^	2.90 ± 0.64^a^	2.70 ± 0.72^b^	4.21 ± 1.5
Differential granulocytes (%)	51.54 ± 11.58^a^	49.50 ± 9.70^b^	48 ± 3.76^b^	57 ± 5.48
Haemoglobin (g/dl)	10.37 ± 1.15^a^	10.64 ± 1.55^a^	11.7 ± 1.01^b^	14 ± 0.9
Packed cell volume (%)	34.66 ± 5.56^a^	32.63 ± 5.14^a^	36.75 ± 1.53^b^	51 ± 3.23
Platelet (×10^3^/μl)	211 ± 68.11^a^	305.9 ± 56.25^b^	310 ± 49.54^b^	320 ± 42
Differential lymphocytes (%)	61.26 ± 16.58^a^	45.50 ± 11.90^b^	49.00 ± 5.66^b^	59 ± 2.53
Eosinophil (μl)	197.01 ± 1.42^a^	177 ± 1.44^b^	132 ± 1.42^c^	242 ± 1.42

Comparison was made between the 1^st^ visit and subsequent visits (treatment period) using ANOVA at a 5% level of significance. Reference values are values obtained from the HIV negative healthy control group. Data with different superscript ^a,b and c^ indicates significant difference at P < 0.05.

A comparative statistical analysis was made between the 1^st^ assessment visit and subsequent visits (treatment period) (Table 2). A consistent reduction in ESR, eosinophil, total white blood cell (WBC), absolute & differential lymphocyte and absolute & differential granulocyte of the patients from the 1^st^ to the 3^rd^ assessment visits was observed with a lower value when compared to the obtained reference values. An exception was noted on ESR which significantly increased on comparison with the reference values. Conversely, we noted a consistent significant (P < 0.05) increase in haemoglobin, platelet and packed cell volume from the 1^st^ to the 3^rd^ assessment visits. All values obtained here was lower than the reference value.

### Biochemical analysis

The concentration of these biochemical parameters; ALP (EC 3.1.3.1; P < 0.05), ALT (EC 2.6.1.2; P < 0.0001), AST (EC 2.6.1.1; P < 0.001), amylase (EC 3.2.1; P > 0.05) and direct bilirubin was significantly increased (upper range) at the third visit though still within the acceptable normal range (Table 3). No remarkable change in plasma creatinine and urea concentrations was observed when compared with the reference values. Albumin levels decreased non-significantly (P > 0.002) when compared with the reference value.

**Table 3:** **Concentration of some biochemical parameters between 1**^**st **^**visit (baseline) and 3**^**rd **^**visit in HIV**^**+ **^**subjects undergoing ART**

**Parameters**	**1**^**st **^**visit**	**3**^**rd **^**visit**	**Reference value**	**P-value**
AST (U/l)	31.66 ± 25.20^a^	34.31 ± 52.21^b^	25 ± 80^c^	P < 0.001
ALT/(U/l)	21.38 ± 14.26^a^	38.47 ± 22.66^b^	20 ± 12.2^a^	P < 0.0001
ALP (U/l)	88.4 ± 21.31^a^	97.31 ± 29.1^b^	62 ± 11^c^	P < 0.05
Amylase (U/l)	59.43 ± 42.62^a^	71.21 ± 41.26^b^	80 ± 25.31^b^	P > 0.05
Plasma urea (mmol/l)	4.17 ± 8.22^a^	4.21 ± 13.64^a^	4.3 ± 10^a^	P < 0.002
Plasma creatinine (mmol/l)	1.20 ± 0.99^a^	1.10 ± 1.01^a^	1.23 ± 0.9^a^	P < 0.629
Direct bilirubin (mg/dl)	0.19 ± 0.10^a^	0.21 ± 0.11^b^	0.18 ± 0.07^a^	P < 0.001
Albumin (U/l)	3.3 ± 2.3^a^	3.02 ± 2.8^a^	4.2 ± 1.1^b^	P < 0.002

Data on 1^st^ and 3^rd^ visits were compared against the reference values using ANOVA at a 5% level of significance. Reference values are values obtained from the HIV negative healthy control group. Data with different superscript ^a,b and c^ indicates significant difference.

### Abnormalities

**Figure 1:**
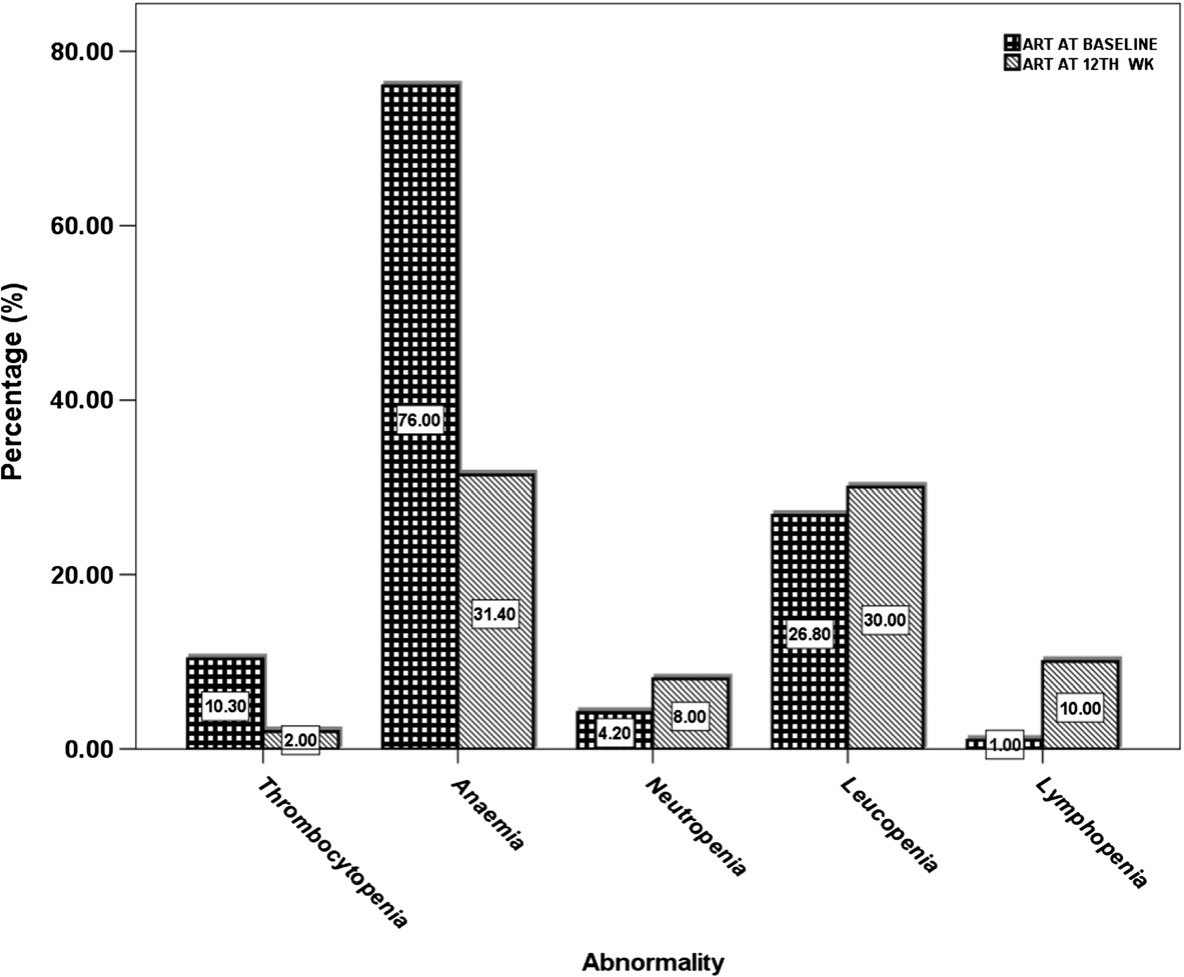
**Distribution of haematologic abnormalities (Cytopenia) observed in HIV**^**+ **^**subjects taking Winniecure ART.** This figure illustrates the distribution of haematological abnormalities (Cytopenia in percentages %) observed in HIV^+^ subjects taking Winniecure ART. The bars compares the abnormality at baseline (1^st^ wk) and at the 12^th^ wk of ART administration. Abnormalities presented include anaemia, neutropenia, leucopenia, lymphopenia and thrombocytopenia.

Various haematological abnormalities maybe associated with administration of antiretroviral drugs. The study reveals a higher incidence of thrombocytopenia (10.30%) and anaemia (76%) in ART HIV^+^ subjects at the first visit. Conversely, these decreased (thrombocytopenia-2% and anaemia-31%) at 3^rd^ assessment visit/12^th^ week (Figure 1). Neutropenia (4.2%), leucopenia (26.8) and lymphopenia (1%) were low at the first visit but showed higher values; 8%, 30% and 10% respectively at the 12^th^ week/3^rd^ visit (Figure 1). No cases of neutrophilia, lymphocytosis, eosinophilia and leukocytosis was observed or associated with the drug administration.

## Discussion and conclusions

The study seeks to evaluate some haematological and biochemical indicators of abnormalities in HIV infected patients administered with Winniecure chemotherapeutic agent used in the treatment of HIV/AIDS in some parts of Nigeria.

HIV infection has been reported to cause diverse degree of immunopathogenesis in man [22] and this carries enormous haematologic and biochemical consequences. The haematologic implications of HIV infection is dominated by peripheral blood cytopenias which, have become more common with the advent of antiretroviral (AR) therapy. Winniecure has been shown to inhibit viral replication and could restore human immunity necessitating its use in the region as anti-HIV medication. As an antiretroviral therapy, it could trigger haematologic consequences as also seen in other AR drugs [23]. Winniecure is a herbal composition of Fiscus exaspirata, Fiscus sur, Sida acuta/Sida corymbosa with a strong hydrophilic nature, moisture content of 0.1% and density 1.936 ± 0.2. It has the following phytochemicals; tannins, saponins and carbohydrates [24]. Previous studies have reported its safety in humans and animals [25].

The baseline characteristics of the study population showed no significant age difference between the reference or control group with that of the test subjects. This implies a reduced variation that could emanate from the pharmacological effect of age on chemotherapeutic agent. The number of HIV infected subjects are generally reported to be higher in the female gender than in the male counterpart [26]. The results obtained here showed a higher number of female subjects on ART when compared with the reference (HIV seronegative subjects) group. HIV/AIDS has so far severely affected more women than men in sub-Saharan Africa. Here females account for almost 57% of adults living with HIV/AIDS. It is known that of all HIV infected youths in the world (5.4 million), over three million are female [27]. This is so because the virus is more easily passed on to young women owing to their immature vaginal tracts and easily torn tissues. Also contributing to this increase is gender inequalities in many tribes in African which, prevent young women from negotiating safer sexual practices that include use of contraceptives such as condom. This influence may account for the increase in the female prevalence rather than the sheer believe on socio-cultural aspects of feminine disposition to attending HIV clinic.

It is generally believed that single/unmarried young individuals are more likely to acquire HIV infection than married partners [28]. Contrarily, the study showed a higher incidence of HIV infection in married partners when compared to single/unmarried individuals, this concurs with the report of other researchers [29,30]. Inasmuch as unmarried/single individuals are likely to engage in multiple sex partners, it is the authors opinion that this group may likely have higher propensity to use protection mechanism against HIV infection than in married individuals with limited sexual partners. Haque and Soonthorndhada [31] previously reported increased STI risk perception and reason for condom use in unmarried young individuals than their married counterpart.

HIV patients assessing ART have been reported to have an increased CD4 cell count, normally initiating treatment from 100-260 cells/μl baseline [32,33]. Starting ART at CD4 cell counts below 50 cells/μl increases the relative risk of death by approximately 60% when compared with ART initiation at 150-249 cells/ul CD4 [34]. Majority (88%) of the study subjects have CD4 cell count between 200-350 cell/ul (i.e. category II based on CDC classification). This shows that the immune status of the subjects fall within the recommended CD4 count for ART initiation (Table 1).

In the course of ART administration, clinically significant haematologic abnormalities maybe common in persons with HIV infection. Impaired haematopoiesis, immune-mediated cytopenias and altered coagulation mechanisms have all been described in HIV-infected individuals. Abnormalities may occur in individuals as a result of the following actions; HIV infection, sequel of HIV-related opportunistic infections, malignancies and consequence of therapies used for HIV infection and associated conditions. In the study findings a significant variation was observed in some of the haematological parameters examined in HIV^+^ subjects on Winniecure treatment (Table 2). The following haematological abnormalities (Figure 1) were thus observed; thrombocytopenia, anaemia, neutropenia, leucopenia and lymphopenia.

Generally, the overall White Blood Cell (WBC) count is important to monitor because elevation of WBC may indicate infection, lack of response to treatment or an abnormality. The total WBC count shows a consistent significant reduction in all the visits although the reduction at the 3rd visit was non-significant (P > 0.05). The progressive reduction (from 1^st^ to 3^rd^ visit) observed in absolute lymphocyte and total WBC may indicate suppressive activity of the antiretroviral drug on the virus with the resultant increase in leucopenia (mild leucopenia; 26.8% to 30%) and lymphocytopenia (moderate lymphocytopenia; 1% to 10%). Leucopenia (a decrease in the number of white blood cells ) and Lymphopenia (decrease in lymphocyts) are the hallmarks of HIV infection and is thought to be mediated by infection of the virus with subsequent killing of CD4+ T cells. It can also be caused by certain medications such as ART [35-37] and certain infections [38,39]. The significant reduction found in the lymphocyte count at the 2^nd^ visit follows the pattern already reported by Stein and his colleagues [40]. However, the transient lymphopenia is common and one-third of the patients may have a typical lymphocytes in the peripheral blood smear. The mechanism responsible for leucopenia and lymphopenia could be an accumulation of Winniecure metabolites (though not determined empirically). We would not de-emphasize the possibility of a pseudoleucopenia since the result showed a mild leucopenia and an increase in Hb as the treatment progresses. Further investigation is needed to ascertain this since the study is devoid of CD4 and viral load assessments hence its limitation.

Several research works have shown that administration of ART especially Zidovudine (AZT) therapy causes anaemia with a significant reduction in Hb in HIV patients [41] even at 6 weeks of administration [42,43]. Interestingly, the result showed an increased PCV at the 2^nd^ and 3^rd^ visits, the data tend to have similar progression with the values obtained from Hb. This suggests an effective therapeutic effect of the drug at the 12^th^ week/3^rd^ visit since a decreased PCV indicates anaemia. The increase at the 3^rd^ visit may indicate a peak active period of the Winniecure treatment within the observation time frame with the resultant reduction of anaemia from 76% to 31%. Recent research works have also shown that mean haemoglobin increases significantly in patients who receive ART thus reversing HIV associated anaemia [44]. The consistent reduction of ESR (41.46 ± 31 to 32.12 ± 15.49) and eosinophil (197.01 ± 1.42 to 132 ± 1.42) from 1^st^ week to 12^th^ week attest to the reduced anaemic condition found in the HIV subjects on Winniecure therapy. Raised ESR and esonophil levels have also been shown to indicate acute anaemic condition [45]. ESR increased significantly when compared with the control, this often may rise significantly in individuals due to infection or medication and merely reflect the anaemic condition seen in these subjects. Moreso, Ndakotsu et al. [46] have reported high ESR in HIV patients of Nigerian origin.

A significant reduction in both differential and absolute granulocyte count was observed between the 1^st^ and 3^rd^ visits. Using the National Cancer Institute-Common Toxicity Criteria (NCI-CTC) Version 2.0 it was found that the subjects undergoing treatment are at NCI risk category 1, implying a mild neutropenia. It is of note that abnormal granulopoiesis and anti-granulocyte antibodies have been noted and described in 30-70% of HIV infected patients [47]. This is believed to contribute to the observed increase in neutropenia (4.2 to 8%). The major clinical significance of the HIV induced neutropenia is that it often precludes therapy with ART and some drugs used for the treatment of opportunistic diseases [48]. The low granulocyte count seen in the 3^rd^ visit may reflect the action of Winniecure on HIV infection or associated conditions. The incidence of neutropenia at the first visit (untreated patients) is consistent with other reports [49] which have also shown a high incidence of granulocytopenia particularly in patients with more profound immunodeficiency.

An escalated and uncontrolled platelet count may indicate disease progression and may sometimes be associated with abnormal bleeding. Thrombocytopenia however, is defined as any disorder in which there is an abnormally low amount of platelets which may result due to immune system malfunction [50]. The result here showed a consistent and significant increase in platelet count from the 1^st^ to the 3^rd^ visits. This indicates a reduced incidence of thrombocytopenia in the HIV^+^ ART subjects. This action maybe adduced to the activity of the administered Winniecure.

Numerous studies have found that liver problems are the most common non-AIDS cause of death among people with HIV infection. Also liver-related death is one of the major causes of mortality accounting for 14-18% of all deaths in the effective antiretroviral therapy era [51-53]. The findings show an elevated liver transaminase enzymes (AST: 34.311 U/L, ALT: 38.47 U/L and ALP: 97.31 U/L) from baseline (1^st^ visit) to the 12 ^th^ week (3^rd^ visit). It is of note that this increase is still within the normal laboratory reference ranges and the result however agrees with the report of Abdullahi et al. [24]. It is our opinion that long term administration of the drug should be guarded to avoid possible drug induced injury as Sulkowski [54] reported that in all the PI regimens studied, the greatest risk of developing drug induced liver injury has been observed among patients on long term therapy receiving full-dose of ritonavir with elevated hepatic enzymes. Furthermore, birirubin a green tetrapyrrolic bile pigment is created by the activity of biliverdin reductase on biliverdin as a product of haeme catabolism. It is also cleared by the liver. The increased direct bilirubin value obtained shows normal conjugation of bilirubin by the liver. Similarly, albumin a protein made specifically by the liver, is slightly decreased (3.02 ± 2.8 U/I) at the 12^th^ week thus indicating a non-significant change.

The pancreatic enzyme amylase (71.21 ± 41.26 U/I) was mildly elevated at the 12th week/3^rd^ visit. Though amylase increased during treatment, it is still within the normal reference range. No case of hyperamylasaemia could be established at the 12^th^ week of administration but there could be possibility of elevated levels at long term treatment. Similarly, plasma creatinine and urea an excellent indicator of kidney function did not show any significant change in their plasma levels between 1^st^ and 3^rd^ visits and with the reference values. This may indicate a normal functional and intact kidney.

In summary the efficacy of the drug has a reduced haematological and biochemical abnormalities with a normal kidney function. The drug seems to may have worked optimally at about 12 weeks of treatment within the period of test thereby indicating appreciative positive changes in the haematological and biochemical indices measured. These results show that the therapeutic efficacy measured on haematological and biochemical responses are accompanied by a reduction of these (parameters measured) indicators of abnormality. They also suggest possible tendency of the administered ART to drive a shift from the physiological normal ranges of haematological and biochemical (mostly liver damage) indices of healthy status to an elevated range.

Admittedly, the study did not consider the intriguing possibility of symptomatic improvement, palliative benefit and CD4 count improvement of the administered drug nor its comparison with any other known antiretroviral drugs thus its limitation.

## Ethical approval

Ethical clearance obtained from Emerging Health and Environment Initiative (EMHEI)/Michael Okpara University of Agriculture with reference N0: Ibeh-2010-25.

## Competing interests

The authors declare that they have no competing interests.

## Authors’ contributions

BI: designed the work, interpreted the results. Laboratory analysis and drafting of the original manuscripts and final approval of the version. OO: involved in the project design, result interpretation and analysis laboratory analysis. UI: critical revision of draft article for suitability and intellectual content, statistical analysis and recruitment of the study population. JH: Draft and final revision of manuscript, statistical analysis and subjects recruitment. All authors read and approved the final manuscript.

## Authors’ information

BI: Holds Ph.D in Medical Biochemistry, a University lecturer and currently is an Assistant Director, Medical Biotechnology Department, National Biotechnology Development Agency Abuja, Nigeria. BI is an active member of Nigerian Society of Biochemistry and Molecular Biology ( NSBMB ) and International Union of Biochemistry and Molecular Biology( IUBMB). An external examiner to two Universities and have published several papers and conference proceedings.

OO: A lecturer at the Department of Biochemistry, Michael Okpara University of Agriculture. A Pharmacology expert and a Medical laboratory Scientists.

UI: Holds Doctor of Optometry (OD). A biostatician and HIV programme specialist at Emerging Health and Environment Initiative.

JB: A Deputy Director at National Biotechnology Development Agency.
